# Intentional Preoperative Weight Loss for Obesity in Patients Undergoing Gastrointestinal Cancer Resections: A Systematic Review and Meta-analysis

**DOI:** 10.1007/s12029-026-01405-1

**Published:** 2026-02-03

**Authors:** Rathin Gosavi, Mehri Anayatullah Rasooli, Noel Leon, Kousitha Sivayogan, Dion Koh, Jason Hong, Vignesh Narasimhan, Geraldine Jia-Ping Ooi

**Affiliations:** 1 Colorectal Unit, Monash Health, Clayton, VIC Australia; 2 Colorectal Unit, Cabrini Hospital, Malvern, VIC Australia; 3 Upper Gastrointestinal Unit, Monash Health, Clayton, VIC Australia; 4 Department of Surgery, School of Clinical Sciences, Faculty of Medicine, Nursing and Health Sciences Monash University, Clayton, VIC 3168 Australia

**Keywords:** Obesity, Preoperative weight loss, Gastrointestinal cancer, Perioperative optimisation

## Abstract

**Background:**

Obesity is increasingly prevalent in patients undergoing gastrointestinal (GI) cancer surgery and is associated with higher rates of intraoperative and postoperative complications. Preoperative weight loss has been proposed as a potential strategy to optimise surgical outcomes, but evidence regarding its safety, efficacy, and feasibility remains unclear.

**Methods:**

A systematic search was conducted across MEDLINE, PubMed, EMBASE, and Cochrane from inception to 28th May 2025, following PRISMA 2020 guidelines and a registered protocol (PROSPERO ID: CRD42020154074). Eligible studies included adults (≥ 18 years) with obesity (BMI ≥ 30 kg/m²) undergoing surgery for GI cancers who received a structured preoperative weight loss intervention. Primary outcomes were feasibility and overall postoperative complication rates. Secondary outcomes included anastomotic leak, operative time, and adverse events. Meta-analysis was performed per outcome measure.

**Results:**

Eight observational cohort studies comprising 532 patients (213 weight loss intervention vs. 319 control) were included. Completion rates for preoperative weight loss were ≥ 96.9%, with no intervention-related harms or surgical delays reported. Pooled analysis demonstrated a significant reduction in overall postoperative complications (OR 0.37, 95% CI 0.16–0.85; *p* = 0.02) and anastomotic leak (OR 0.26, 95% CI 0.12–0.60; *p* = 0.002) in the intervention group. Where assessed, skeletal muscle mass and nutritional parameters were preserved.

**Conclusion:**

Intentional preoperative weight loss appears feasible, safe, and may improve postoperative outcomes in patients with obesity undergoing GI cancer surgery. Integration of such strategies, particularly during neoadjuvant therapy windows, offers a modifiable opportunity to optimise surgical risk. Prospective trials are warranted to define optimal protocols, timing, and oncologic safety.

**Supplementary Information:**

The online version contains supplementary material available at 10.1007/s12029-026-01405-1.

## Introduction

Obesity has reached epidemic proportions worldwide, with more than 35% of adults in Australia now classified as obese [1]. This rising prevalence is increasingly mirrored in cancer populations, where obesity is not only common but also contributes to oncogenesis. Adiposity is a recognised risk factor for the development of several gastrointestinal malignancies, including colorectal, pancreatic, oesophageal, and gastric cancers [2]. It not only raises the risk of cancer-related mortality but also influences treatment options. Estimates suggest that obesity accounts for approximately 4–8% of cancer cases globally [2]. We are now faced with a growing proportion of patients presenting for major gastrointestinal cancer surgery who have concurrent obesity or have obesity-related metabolic comorbidities.

Obesity adversely impacts perioperative outcomes across a range of surgical disciplines, and its effect is particularly pronounced in cancer surgery. Elevated risks of surgical site infection, anastomotic leak, cardiopulmonary complications, and prolonged hospitalisation have been consistently reported in people with obesity undergoing colorectal, gastric, and hepatobiliary resections [3–5]. Beyond the mechanical challenges of operating in visceral obesity, systemic inflammation, impaired glycaemic control, and reduced cardiorespiratory reserve likely compound surgical risk and impair recovery [6, 7]. Despite this, there are few recommendations or guidelines informing the management of obesity in patients undergoing cancer surgery.

Preoperative weight loss represents a potentially modifiable risk factor. Even modest reductions in weight (e.g. 5–10% total body weight loss (TBWL)) may improve physical function, enhance cardiorespiratory fitness, and reduce systemic inflammation in benign conditions [8]. In the elective general and bariatric surgery literature, preoperative weight loss is associated with fewer postoperative complications, shorter length of stay, and improved functional recovery [9, 10]. Whether these benefits extend to patients undergoing gastrointestinal cancer resection remains uncertain.

However, weight loss in the context of obesity and cancer care is complex. Many patients experience unintentional weight loss, with associated sarcopaenia and functional decline at the time of diagnosis [11]. Psychological distress, and the commencement of systemic therapies such as chemotherapy or radiotherapy can further hinder engagement in weight optimisation interventions. Moreover, obesity, itself, is accompanied by stigma and negative preconceptions, and can lead to reduced participation [12]. These competing considerations make intentional, structured weight loss interventions an area of clinical uncertainty in GI cancer surgery.

This systematic review aims to critically evaluate the available evidence on the feasibility, safety, and perioperative impact of intentional preoperative weight loss programs in patients with obesity undergoing gastrointestinal and hepatobiliary cancer surgery. Where possible, pooled analysis of postoperative complications and anastomotic leak will be performed to quantify the potential benefit of these interventions.

## Methods

For this review, we focused on studies that implemented intentional weight loss measures for patients with obesity who were planned to undergo curative-intent abdominal surgery for gastrointestinal cancer.

This systematic review was performed using the Preferred Reporting Items for Systematic review and Meta-Analysis Protocols (PRISMA-P) guidelines. It was registered on PROSPERO (CRD42020154074) on 26th May 2025.

### Search Strategy

A preliminary scoping search was conducted in MEDLINE, PubMed, and Cochrane Library databases to identify relevant keywords and subject headings appearing in titles, abstracts, and indexing terms. Subsequently, a comprehensive search of the MEDLINE, PubMed, Embase and the Cochrane Library databases was performed from inception to 28th May 2025. The search strategy incorporated terms related to obesity, weight loss, cancer surgery, and gastrointestinal or hepatobiliary malignancy. Key search terms included “obesity,” “weight loss,” “preoperative,” “bariatric,” “very low-calorie diet,” “GLP-1”, “cancer surgery,” “colorectal,” “gastric,” “oesophageal,” “pancreatic,” “liver,” “hepatic,” and “biliary.” The reference lists of included studies were also manually screened to identify any additional relevant publications. The full search strategy is found in Supplementary Materials.

### Study selection

**Fig. 1:**
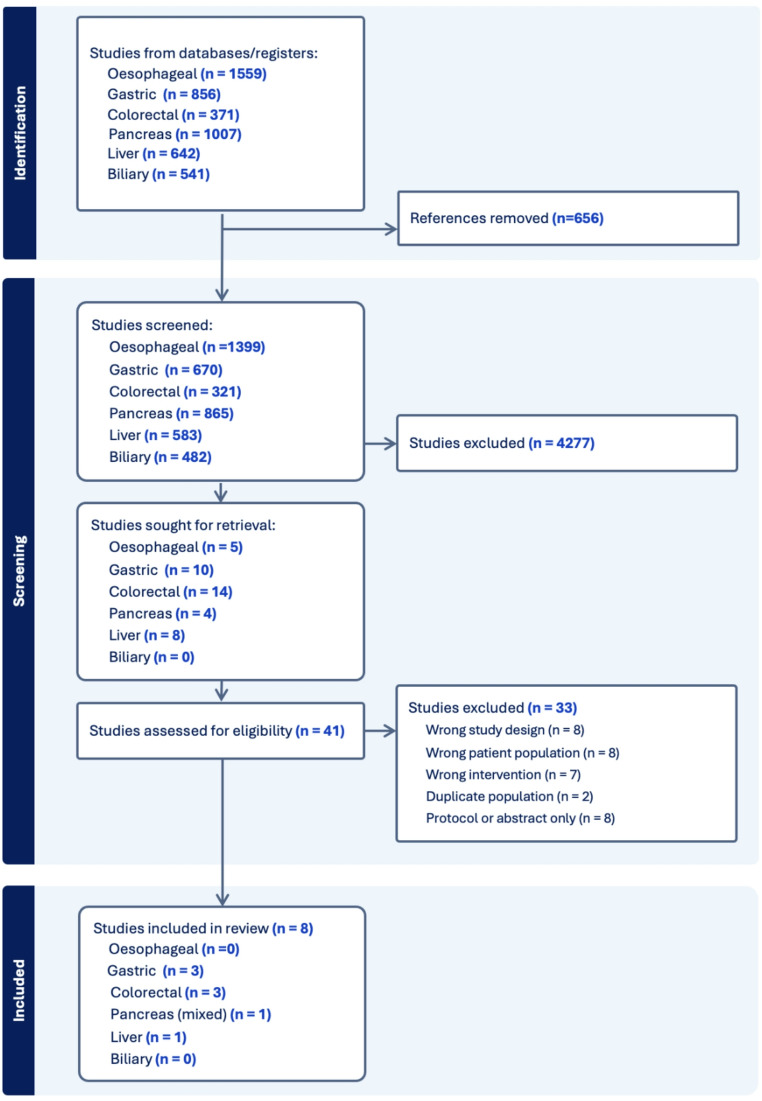
PRISMA chart of study selection

All identified records were imported into Covidence (Veritas Health Innovation, Melbourne, Australia). Covidence system duplicate detection was used to remove duplicates and duplicates were verified by NL. Title and abstract screening, followed by full-text review, was conducted independently by two reviewers within the Covidence platform. Discrepancies were resolved through discussion or adjudication by the supervising reviewer, GO. The selection process is presented in a PRISMA 2020 flow diagram (Fig. 1).

### Eligibility Criteria

For this review, studies had to meet al.l of the following inclusion criteria: (1) patients with obesity, defined as a body mass index (BMI) of ≥30 kg/m² in Western cohorts or ≥25 kg/m² in Asian cohorts; (2) adult patients ≥18 years old; (3) undergoing curative intent elective abdominal surgery for a histologically confirmed gastrointestinal cancer; (4) undergoing a preoperative intentional weight loss intervention (WLI), including but not limited to calorie-restricted diet, supervised exercise program, multimodal prehabilitation protocol, or metabolic bariatric surgery (MBS); (5) reported on postoperative outcomes. Eligible study designs included retrospective and prospective cohort studies, matched case-control studies, and other comparative observational designs. We included studies without a control arm in order to report widely on methodology, safety and feasibility outcomes for obesity interventions in this population, although these results were unable to be pooled.

Exclusion criteria were: (1) non-English based publications; (2) studies involving peritonectomy, cytoreductive surgery, intraperitoneal chemotherapy; (3) studies focused on unintentional weight loss; (4) case series ≤ 5 patients; (5) conference abstracts, unpublished works or review articles.

### Data Extraction

Data were extracted independently by two reviewers using a standardised spreadsheet to capture key study characteristics and outcomes. Extracted data included study design, sample size, patient demographics, cancer type, BMI, intervention type and duration, degree of weight loss, and reported postoperative outcomes. Feasibility metrics (including adherence and surgical delay), complication rates, anastomotic leak, operative time, length of stay, and estimated blood loss were collected where available. Discrepancies were resolved through discussion or adjudication by a third reviewer. Where outcomes were reported as medians with interquartile ranges or minimum–maximum values, means and standard deviations were estimated using validated formulae proposed by Wan and colleagues [13].

### Outcomes

The primary outcome was rate of any postoperative complication. Secondary outcomes included specific postoperative outcomes, such as surgical site infection (SSI), anastomotic leak, bleeding, fistula rates and length of stay, and intraoperative variables including operative time and complications. We collected data on issues around safety, compliance, completion and tolerability of weight loss intervention (WLI), as well as efficacy in achieving weight loss.

### Subgroup Analyses

We prespecified exploratory subgroup analyses by cancer site (colorectal vs. gastric). Subgroups with at least three contributing studies were meta-analysed; where fewer than three studies were available or clinical heterogeneity was substantial, findings were summarised narratively and presented in the supplement without pooling.

### Risk of Bias Assessment

Risk of bias for each included study was assessed using the Cochrane ROBINS-I Version 2.0 (2024 launch version) tool. The tool evaluates seven domains of potential bias, including confounding, selection of participants, classification of interventions, deviations from intended interventions, missing data, measurement of outcomes, and selection of the reported result. Each domain was rated as having low, moderate, serious, or critical risk of bias. Disagreements were resolved through discussion or by adjudication by the supervising reviewer, GO. Risk of bias assessments were summarised narratively and presented in tabular form.

### Data Synthesis and Statistical Analysis

Dichotomous outcomes were pooled using the Mantel–Haenszel (MH) random-effects model (DerSimonian–Laird) and reported as odds ratios (ORs) with 95% confidence intervals (CIs). Because anastomotic leak events were rare, the primary leak analysis used the Peto fixed-effect method; as Peto assumes balanced allocation and sparse events, estimates were interpreted cautiously. Continuous outcomes were pooled as mean differences under random-effects. Heterogeneity was assessed with the I² statistic. A pre-specified exploratory cancer-site subgroup (colorectal vs. gastric) was undertaken as above; where < 3 studies were available or clinical heterogeneity was substantial, results were summarised narratively and shown only in the supplement. Sensitivity analyses included the Hartung–Knapp–Sidik–Jonkman adjustment and exclusion of the largest study. Two-sided p values < 0.05 were considered statistically significant. Analyses were performed in RevMan Web (version 5.4).

## Results

### Study Selection

A total of 4,976 records were retrieved from electronic databases across six cancer streams: oesophageal (*n* = 1,559), gastric (*n* = 856), colorectal (*n* = 371), pancreatic (*n* = 1007), biliary (*n* = 541), and liver (*n* = 642). After removal of 656 duplicate records, 4320 records remained for screening by title and abstract.

Following title and abstract screening, 4277 records were excluded as irrelevant. 41 full-text articles were reviewed in detail across the six tumour types. Ultimately, eight studies met the inclusion criteria [14–21]. No eligible studies were identified for the oesophageal or biliary cancer streams. The study selection process is summarised in Fig. 1.

### Study Characteristics

Eight observational cohort studies comprising a total of 532 patients (213 WLI vs. 319 control) were included in the final analysis (Table 1). The studies were published between 2014 and 2024 and originated from Japan (*n* = 6) [14–19], Canada (*n* = 1) [20], and multisite (US, UK) (*n* = 1) [21]. Cancer types included colorectal (*n* = 3), gastric (*n* = 3), mixed GI (*n* = 1), and hepatocellular carcinoma (*n* = 1).

**Table 1:** Baseline characteristics of studies

Study	Study type	*n*= (WLI/control)	Age/Gender*	Weight/BMI*	Operations*	Disease stage*
Parmar 2024 Multicentre (Mixed)	Multisite, retrospective cohort	13 GI tumours (WLI only): 6 colorectal, 6 pancreas,1 small bowel	(Any tumour, *n* = 37) 52 years (IQR 40.5–60) 9 male/28 female	(GI tumours, *n* = 13) 46.8 kg/m^2^ (IQR 41.6–52.6, range 40–69.6.6)	Whipples (2), total pancreatectomy/spleen (2), extended distal pancreatectomy (1), distal pancreatectomy (1), colectomy (2), LAR (3), total proctectomy (1)	Pancreatic NET: Stage 1 (2), Stage 3 (1) - Pancreas IPMN: Stage 1 (2) - Pancreas intraepithelial neoplasm (1) - Colorectal: Stage 1 (2), Stage 2 (1), Stage 3 (2) - Small bowel NET: NR
Saito 2023 Japan (Liver)	Single site, retrospective cohort, propensity matched	19 (WLI) Propensity matched analysis: 16 (WLI) vs. 16 (control)	74 years (59–83) 12 male/4 female	25.8 kg/m^2^ (range 25.1–30.8)	Extent: Hr0 8 (5 WLI vs. 3 none), HrS 7 (4 vs. 3), Hr1 13 (5 vs. 8), Hr2 4 (2 vs. 2) Open/laparoscopic: 15/1	Stage I (2), Stage II (9), Stage III (5) Tumour size (cm): 3.5 (1.5–11.0)
Kashihara 2021 Japan (Gastric)	Single site, prospective cohort	8 (WLI) vs. 14 (retrospective control)	69.8 +/- 7.5 years 6 male/2 female	30.1 +/- 7.9 kg/m^2^ (range 28.0–34.8.0.8) 73.1 +/- 6.3 kg (range 58.5–81.7)	Lap distal gastrectomy (4), lap total gastrectomy (3), lap PPG (1)	Stage I (7), Stage II (1)
Inoue 2018 Japan (Gastric)	Multisite, prospective cohort	33 (WLI) vs. 23 (retrospective control)	71 years (range 41–81) 26 male/7 female	26.0 kg/m^2^ (23.5–31.0) 72.3 kg (range 53.8–82.5	Total gastrectomy (3), distal gastrectomy (27), PPG (2), proximal gastrectomy with oesophagogastrostomy (1)	pStage I (27), pStage II (3), pStage III (3)
Cho 2014 Japan (Gastric)	Prospective matched pair cohort	18 (WLI) vs. 54 (1:3 matched control)	63.1 years (range 51–76) 18 male	26.7 kg/m^2^ (range 23.1–31.2)	Total gastrectomy (4), distal gastrectomy (14) Open/lap: 9/9	pStage IA (16), pStage IB (2)
Nakagawa 2020 Japan (Colorectal)	Single site, retrospective/prospective cohort	26 (WLI only)	60.6 +/- 12.4 years 15 male/11 female	30.4 +/- 4.7 kg/m^2^ 79.8 +/- 15.6 kg	LAR (11), HAR (3), colectomy (6), CAA (4), intersphincteric resection (1), ileocolic resection (1)	pStage 0 (1), pStage I (16), pStage II (2), pStage III (7)
Kashihara 2021 Japan (Colorectal)	Single site, prospective cohort	7 (WLI) vs. 113 (retrospective control)	65.7 years (range 53–80) 3 male/4 female	33.9 kg/m^2^ (range 30.7–43.7) 87.0 kg (range 73.9–109)	Ileocolic resection (1), colectomy (4), HAR (2)	Stage I (6), Stage II (1)
McKechnie 2024 Canada (Colorectal)	Single site, retrospective cohort	89 (WLI) vs. 101 (retrospective control)	66 years (IQR 58–72) 43 female	35.9 kg/m^2^ (33.2–39.4) 102.1 +/- 16.9 kg	(All WLI patients, *n* = 89) Right hemicolectomy (27), left hemicolectomy (4), anterior resection (24), LAR (26), total colectomy (1), total proctocolectomy (1), ileostomy reversal (3), Hartmann reversal (3)	(WLI patients with cancer, *n* = 62): Stage I (23), Stage II (13), Stage III (22), Stage IV (3), NR (3)

*BMI* body mass index; *CAA* coloanal anastomosis; *HAR *high anterior resection; *Hr0* nonanatomical resection; *HrS* single segment; *Hr1* 1 section (left lateral, left medial, right anterior, right posterior); *Hr2* 2 sections (right hemihepatectomy; *IPMN* intraductal papillary mucinous neoplasm; *IQR* interquartile range; left hemihepatectomy, central bisectionectomy); *Hr3* 3 sections (right trisectionectomy, left trisectionectomy); *lap* laparoscopic; *LAR* low anterior resection; *LDG* laparoscopic distal gastrectomy; *LTG* laparoscopic total gastrectomy; *NET* neuroendocrine tumour; *NR* not reported; *PPG* pylorus-preserving gastrectomy; *WLI* weight loss intervention. *Age/gender/wt/BMI/operations/disease stage reported for WLI only

Preoperative weight loss interventions varied in modality and duration but included very low-calorie diets (n_study_=6, n_participants_=179) [15–20], supervised exercise programs (n_study_=5, n_participants_=75) [14, 16–19], and, in one study, pre-treatment metabolic bariatric surgery (n_study_=1, n_participants_=13) [21]. Baseline BMI ranged from 25.8 to 46.8 kg/m² across studies.

All except two studies examined early cancer with participants that did not require neoadjuvant therapy. McKechnie et al. [20] included colorectal cancer of any stage, with 14 participants undergoing WLI having concurrent neoadjuvant therapy (15.7%). There were no withdrawals from this group. Parmar et al. [21] included nine patients who had neoadjuvant therapy after metabolic bariatric surgery.

### Weight Loss Intervention

#### Feasibility and Safety

Feasibility was reported across all eight included studies. Completion rates for the preoperative weight loss intervention ranged from 96.9 to 100%, with no study reporting attrition that resulted in surgical delay. Of all the studies, only one patient in study by Inoue et al. [15] ceased preoperative VLED due to product taste (1 of 33, 3.1%) (Table 2).

**Table 2:** Obesity/weight loss intervention implemented by study

Study	Target cancer group	*N*=		Intervention	Supervision	Duration	Dropout	Neoadjuvant therapy
		WLI	Control					
Parmar 2024 Multicentre (Mixed)	Pancreas (6), colorectal (6), small bowel (1)	13	None	Bariatric surgery: SG (7 panc, 3 CRC), RYGB (1 SB, 1 CRC), OAGB (1 CRC)	NR.	6.9 +/- 4.2 months	NA	Chemoradiotherapy (4), chemotherapy (4), hormonal therapy (1) (after MBS)
Saito 2023 Japan (Liver)	HCC	16	16	Caloric restriction (SBW x 20 kcal/day) Resistance/aerobic exercise training (5 days/week, 60–120 min sessions)	Dietician and physical therapist. Support throughout (details not specified).	‘Time of admission’ until surgery. 21 +/- 12 days	NR.	NR.
Kashihara 2021 Japan (Gastric)	Early gastric (cT1a-b)	8	14	Caloric restriction (1200 kcal/day) Tailored exercise (resistance and aerobic)	Rehabilitation department. Otherwise not stated.	10–30 days pre-op 26.3 +/- 6.6 days (range 19–40)	NR.	NR.
Inoue 2018 Japan (Gastric)	Stage I gastric cancer (cT1N0-1/cT2N0)	33	23	Nutritional counselling by dietician. VLED replacing one meal/day.	Initial consultation and 1x follow-up.	20 days pre-op	*n* = 1 (3.1%), due to taste	NR.
Cho 2014 Japan (Gastric)	Stage I gastric cancer (cT1N0-1/T2N0)	18	54	Aerobic exercise/stretching (3–7 days/week), resistance training (1–2 days/week). Energy expenditure 30 kcal/kg/week.	NR.	4 weeks pre-op	None	NR.
Nakagawa 2020 Japan (Colorectal)	Stage 0-IIIb colorectal cancer	26	None	Dietary guidance. Jogging 60 min/day.	Initial education by nurses, dieticians and physiotherapists. Nurse-led supervision.	1 week post-initial consultation to day of admission.	None.	Excluded if receiving chemotherapy or radiotherapy.
Kashihara 2021 Japan (Colorectal)	Early colorectal cancer (≤ T2)	7	113	Caloric restriction (1200 kcal/day). Tailored exercise (resistance and aerobic)	Rehabilitation department. Otherwise not stated.	Preoperative. 29.6 days (15–70)	None.	NR.
McKechnie 2024 Canada (Colorectal)	Colorectal resections (includes malignant (any stage) and benign)	89	101	VLED: QID Optifast 900 kcal/day	Not specifically stated - Per local bariatric surgery program.	2–4 weeks pre-op.	None.	Total: *n* = 29 (20.1%) WLI: *n* = 14 (15.7%) Control: *n* = 11 (10.9%)

*CRC* colorectal cancer; *NA* not applicable; *NR* not reported; *OAGB* one anastomosis gastric bypass; pre-op – preoperative; *RYGB* Roux-en-Y gastric bypass; *SB* small bowel; *SBW* standard body weight; *SG* sleeve gastrectomy; *VLED* very low energy diet;

There were no adverse outcomes reported in any studies. Those that reported on body composition measures showed retained skeletal muscle [15, 17–19], hand grip strength [19] and albumin levels [15, 17–19].

Only Parmar et al. [21], which evaluated pre-treatment metabolic bariatric surgery (MBS) in a select cohort, reported a time from diagnosis to resection exceeding three months in all cases. However, all patients had low-grade or indolent tumours, and all underwent cancer-directed therapy as planned, with oncologic surgery becoming subsequently feasible due to MBS surgery. They reported no early (< 30 days) or late (≥ 30 days) MBS-related complications over a mean follow-up of 4.3 ± 3.9 years, and no MBS-related death.

No study reported early termination or program-related adverse events. Where assessed, adherence to dietary targets and attendance at sessions was high. All interventions were completed within a preoperative timeframe, and no study reported breach of oncological resectability windows.

#### Efficacy

Intervention durations ranged from a mean of 21–45.5 days (range 10–121 days) for non-surgical intervention, with mean weight loss ranging from 4.2 to 6.9% TBWL, depending on the modality and intensity of the program. Control groups consisted of either historical or contemporaneously matched patients receiving standard preoperative care without structured weight loss intervention. The one study using MBS surgery [21] achieved a weight loss of 16.4% TBWL (13.2–19.1%) at 3 months post-intervention (Table 3).

**Table 3:** Outcomes of weight loss intervention and cancer surgery

Study	Outcomes from intervention			Surgical outcomes (WLI vs. control)		
	Safety issues	Com-pliance	Weight loss (pre- vs. post-intervention)	Overall and specific complications	Bleeding	Admission and operation
Parmar 2024 Multicentre (Mixed)	No early (< 30 days) or late (4.3 ± 3.9 year follow-up)	NA	Baseline - BMI 48.4; 3 month - BMI 42.6, TBWL 16.4% (*p* < 0.001); 12 month - BMI 33.5, TBWL 31.3% (*p* < 0.001) (*n* = 13 GI cancer): Baseline BMI 46.8; pre-resection BMI 37.3	Any: 2/13 C.diff infection: 1/13	Severe haemorrhage (1/13)	
Saito 2023 Japan (Liver)	NR.	100%	BMI: 28.0 vs. 26.4 (*p* < 0.05) VAT (cm^2^): 171.2 vs. 156.2, *p* < 0.05 SMM (kg/m^2^): 7.28 vs. 7.26, p = n.s. HGS (kg): 29.9 vs. 29.7, p = n.s.	Any: 2/16 vs. 1/16, *p* = 0.54 CD3+: 0 vs. 0	EBL (ml): 141 vs. 200, *p* = 0.24	Op time (mins): 284 vs. 284, *p* = 0.99 LOS: 15 vs. 15 days, *p* = 0.75
Kashihara 2021 Japan (Gastric)	None.	NR.	TBWL 4.2%; WtΔ −3.1 kg (75 vs. 72) (*p* < 0.05); BMI 30.1 vs. 29 (p = n.s); VAT area − 10.6% (*p* < 0.05); Body fat mass − 5.8% (*p* < 0.05); SMM (p = n.s.)	CD3+: 1/8 vs. 5/14, *p* < 0.05 Pancreatic fistula: 0/8 vs. 5/14	EBL (ml): 75 vs. 225, *p* < 0.05	Op time (mins): 320 vs. 425, *p* < 0.05 LOS (days): 15 vs. 19, *p* < 0.05
Inoue 2018 Japan (Gastric)	None	96.9%	TBWL 4.5% (*p* < 0.0001); WtΔ −3.2 kg (95% CI 2.7–3.7) (*p* < 0.0001); BMIΔ − 1.2 kg/m^2^ (*p* < 0.001); SMMΔ − 0.20 kg (*p* = 0.25), VAT (cm^2^) 146.5-118.4.5.4.5.4.5.4 (−16.8%) (*p* < 0.0001); SAT mass − 1.4% (*p* = 0.86)	Operative morbidity: 7 (25.9%) vs. 5 (21.7%), *p* = 0.68	EBL (ml): 49 vs. 75, *p* = 0.043	Op time (min): 355 vs. 355, *p* = 0.35 No conversions in the WLI group (0/33)
Cho 2014 Japan (Gastric)	None.	NR.	BMIΔ − 0.48 (*p* = 0.004); WtΔ −1.34 kg (*p* = 0.004); SATΔ (cm^2^) −9.3 (*p* = 0.302); VATΔ (cm^2^) −34.8 (*p* = 0.025)	Any 5.6% vs. 40.7% (p-0.008); Grade 2 + 5.6% vs. 29.6% (*p* = 0.063); Respiratory (any) 16.7% vs. 14.8% (*p* = 1.000); Wound infection 0% vs. 16.7% (*p* = 0.125); Deep abscess 0% vs. 18.5% (*p* = 0.089); Anastomotic leak 0% vs. 16.7% (*p* = 0.125); Pancreatic fistula 5.6% vs. 14.8% (*p* = 0.565)	EBL (ml): 105 vs. 130, *p* = 0.692 Bleeding: 0% vs. 3.7%, *p* = 1.000	Op time (min): 248 vs. 208, *p* = 0.185
Nakagawa 2020 Japan (Colorectal)	NR.	100%	Wt (kg) 79.8 vs. 75.7 (*p* < 0.05); WtΔ 4.9%±3.4 (*p* < 0.05)	SSI: 5/26 (19.2%)		
Kashihara 2021 Japan (Colorectal)	NR.	100%	Wt (kg) 87.0 vs. 81.0 (*p* < 0.05) BMI (kg/m^2^) 33.9 vs. 31.6 (*p* < 0.05) VAT (cm^2^) 169.0 vs. 138.6 (*p* < 0.05) Body fat mass % 40.7 vs. 36.9 (*p* < 0.05) Skeletal muscle % 31.8 vs. 31.1 (*p* = 0.26)	CD3+: 0 vs. 16%, *p* < 0.05 Anastomotic leak: 0 vs. 8%, *p* < 0.05		Op time (min): 237.9 vs. 231.5, *p* = 0.92 LOS (days): 15.0 vs. 14.0, *p* = 0.56
McKechnie 2024 Canada (Colorectal)	NR.	100%	NR.	Overall morbidity 38.2% vs. 69.3%, *p* < 0.01 Multivariate: Decreased risk of complication with VLED: OR 0.22, 95% CI 0.08–0.61 (*p* < 0.01); Anastomotic leak 2.3% vs. 13.7%; Respiratory 7.9% vs. 20.8% (*p* < 0.01); CVS 6.7% vs. 13.9% (*p* = 0.02); VTE 0 vs. 2.0% (*p* = 0.18); Infection 9.0% vs. 20.8% (*p* = 0.02); Wound 5.6% vs. 16.8% (*p* = 0.02); Reoperation 3.4% vs. 8.9% (*p* = 0.51); Intraoperative Cx 9.0% vs. 16.8% (*p* < 0.05)	EBL: 100 vs. 100, *p* = 0.91	Op time (min): 190 vs. 179, *p* = 0.55 LOS (days): 4 (3–5) vs. 4 (3.7), *p* = 0.13 Conversion 0 vs. 6 (6.5%) (*p* = 0.01)

*CD* Clavien-Dindo complication; *CVS* cardiovascular system; *Cx* complication; *EBL* estimated blood loss; *HGS* hand grip strength; *LOS* length of stay; *MBS* metabolic and bariatric surgery; *NA* not appliable; *NR* not reported; *n.s.* not significant; *OAGB* one anastomosis gastric bypass; *RYGB* Roux-en-Y gastric bypass; *SAT* subcutaneous adipose tissue; *SBW* standard body weight; *SG* sleeve gastrectomy; *SMM* Skeletal muscle mass; SSI – surgical site infection; *TBWL* total body weight loss; *VAT* visceral adipose tissue; *VLED* very low energy diet; *VTE* venous thromboembolism; *WLI* weight loss intervention

Some studies reported on body composition changes, showing significant improvement in visceral fat area [14, 15, 17–19], with − 15 to −34.8cm^2^ reduction reported.

### Postoperative Outcomes

#### Overall Complications

**Fig. 2:**
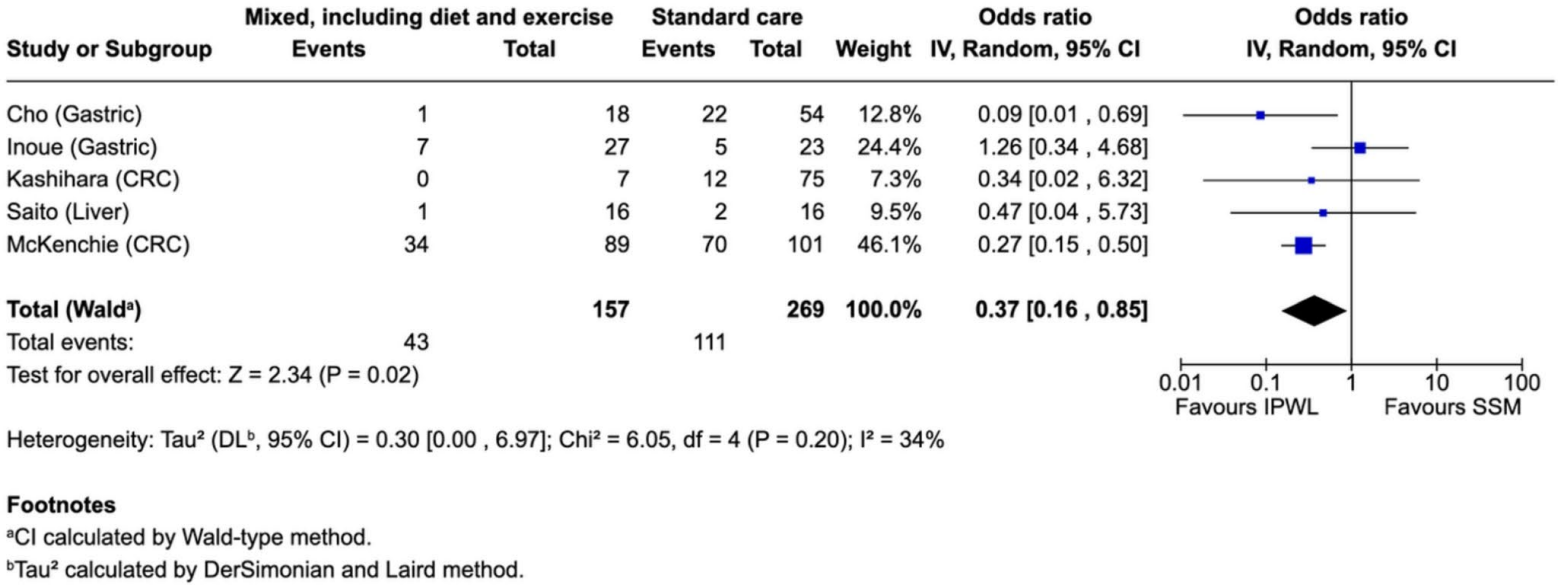
Overall postoperative complication – weight loss intervention vs. control

Overall postoperative complication rates were reported in seven of the eight included studies. Using the MH random-effects model, pooled analysis demonstrated a lower rate of overall postoperative complications in patients undergoing intentional preoperative weight loss compared to controls (OR 0.37, 95% CI 0.16–0.85; I² = 34%; *p* = 0.02) (Fig. 2). The effect direction was consistent across both upper and lower gastrointestinal cancer cohorts. Where reported, the intervention group had either comparable or reduced rates of major (Clavien-Dindo III–IV) complications.

In sensitivity analysis, findings were robust in direction but less precise. Using the Hartung–Knapp–Sidik–Jonkman (HKSJ) adjustment, the pooled effect for overall complications remained in favour of intentional preoperative weight loss (OR 0.37, 95% CI 0.12–1.16; *p* = 0.07; I²=34%; Supplementary Fig. 1). Excluding the largest study (McKechnie et al.) yielded a similar point estimate with wider confidence intervals (OR 0.43, 95% CI 0.11–1.65; *p* = 0.22; I²=40%; Supplementary Fig. 2). Thus, the direction of effect was unchanged across sensitivity analyses, while statistical significance was attenuated, consistent with smaller information size.

#### Subgroup by Cancer Site

In exploratory analyses, the colorectal subgroup showed a significant reduction in overall complications (MH random-effects OR 0.28, 95% CI 0.15–0.50; I²=0%; *p* < 0.001), whereas the gastric subgroup was imprecise and non-significant (OR 0.37, 95% CI 0.02–5.70; I²=80; *p* = 0.48) (Supplementary Fig. 3).

#### Anastomotic Leak

**Fig. 3:**
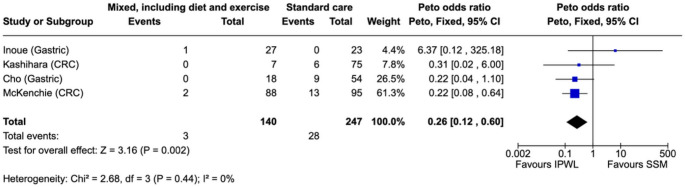
Anastomotic leak – weight loss intervention vs. control

Anastomotic leak was reported in four studies involving colorectal and gastric cancer patients and was pooled using the Peto fixed-effect method due to low event rates, demonstrating lower leak rates in the intervention group (OR 0.26, 95% CI 0.12–0.60; I² = 0%; *p* = 0.002) (Fig. 3). Absolute rates ranged from 0% to 17% across studies, with lower rates observed in the weight loss groups.

In exploratory analysis, the colorectal subgroup showed a significant reduction in leak (OR 0.23, 95% CI 0.09–0.62; I²=0%; *p* = 0.004), while the gastric subgroup was non-significant with wide CIs (OR 0.36, 95% CI 0.08–1.58; I²=58%; *p* = 0.18). Event counts were very low, and these analyses are exploratory (Supplementary Fig. 4).

#### Blood Loss

Estimated blood loss was reported in five studies. One study presented EBL only as a bar chart without extractable numerics and was excluded from pooling [22]. Across the remaining four studies, pooled analysis showed no clear difference between groups (Supplementary Fig. 5).

#### Operative time

Operative time was available in five studies. The pooled estimate across all tumour sites (Supplementary Fig. 6) and by cancer type (Supplementary Fig. 7) showed no clear difference between groups.

#### Other Outcomes

None of the included studies reported a statistically significant increase in reoperation, or readmission in the intervention arm. Thirty-day mortality was reported in three studies and was 0% across all groups.

### Risk of Bias Assessment

**Fig. 4:**
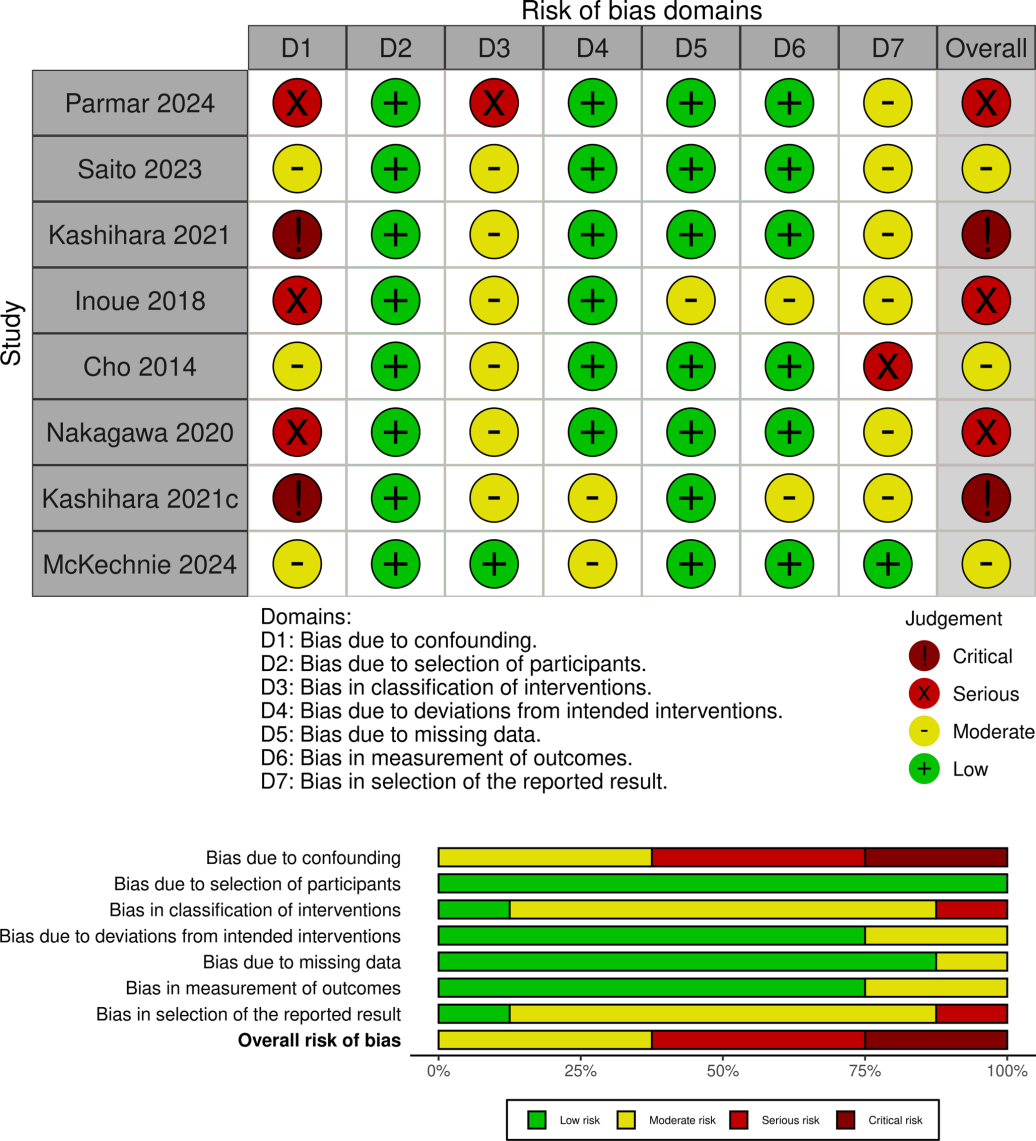
Risk of bias assessment

Risk of bias was assessed using the Cochrane ROBINS-I Version 2.0 tool. All included studies were non-randomised observational cohorts (prospective or retrospective) and therefore at risk of bias from confounding and selection. Most studies demonstrated adequate baseline comparability between groups but were judged at high risk for selection bias due to non-random allocation. Blinding of outcome assessors was not reported in any study. Attrition bias and selective reporting were low across the majority of studies, and postoperative outcomes were generally well defined and consistently reported. Overall, three studies were rated as moderate risk of bias, three were rated as high risk, and two were rated as critical risk, primarily due to confounding, classification of interventions and potential for selection bias (Fig. 4).

## Discussion

This systematic review demonstrates that intentional preoperative weight loss in patients with obesity undergoing gastrointestinal cancer surgery is feasible, safe, and may be associated with improved postoperative outcomes. Across eight observational cohort studies (prospective or retrospective) encompassing a range of malignancies, including colorectal, gastric, hepatic, and pancreatic cancers, completion rates were high. No intervention-related harms were reported, and no delays to surgery were observed in standard care pathways. Where studied, weight loss interventions were associated with lower rates of overall postoperative complications and anastomotic leak.

Obesity presents significant challenges in the surgical management of gastrointestinal cancers. Visceral adiposity complicates anatomical dissection, limits operative exposure, and increases the technical difficulty of achieving safe resection, especially in laparoscopic or minimally invasive approaches. This has been associated with increased operative time, greater blood loss, and higher rates of intraoperative complications such as bowel or vascular injury [23, 24]. Furthermore, obesity-related chronic low-grade inflammation and immune dysfunction—including impaired innate responses (e.g., neutrophil chemotaxis/phagocytosis, macrophage polarisation) and adaptive responses (T-cell dysregulation), together with insulin resistance and impaired microvascular perfusion, increase susceptibility to postoperative wound infection, anastomotic dehiscence, and delayed recovery [25–27]. In this review, pooled data demonstrated a significantly lower rate of overall complications (OR 0.37, *p* = 0.02) and anastomotic leak (OR 0.26, *p* = 0.002) among patients who underwent structured preoperative weight loss, suggesting that any improvements in body composition may yield clinically meaningful benefit.

A variety of interventions were found to be both safe and feasible. These included caloric restriction (with or without formula diets), structured exercise prehabilitation, and multidisciplinary dietary counselling. Interventions were generally completed within preoperative windows of 3–6 weeks, with high adherence and no evidence of nutritional compromise or oncologic delay. However, nutritional status was not systematically assessed across all cohorts; several studies focused on metabolic or functional measures rather than comprehensive nutritional endpoints. Accordingly, the absence of a signal for nutritional compromise should be interpreted cautiously. Notably, McKechnie et al. [20] demonstrated the feasibility of such programs even in patients who had received neoadjuvant therapy, supporting their potential integration in modern multimodal treatment pathways.

Incorporating pharmacological agents such as GLP-1 receptor agonists (e.g., semaglutide and tirzepatide) represents a novel adjunct to non-surgical weight loss. Although not studied in the included trials, these agents are gaining traction in the broader obesity literature for their efficacy in achieving sustained weight reduction, often in combination with dietary modification and physical activity [28, 29]. Their future evaluation in the perioperative cancer setting is warranted, particularly in patients who are unable to achieve weight loss through behavioural interventions alone.

Several gastrointestinal cancers routinely involve a window of opportunity for obesity optimisation. Rectal, oesophageal, gastric, and borderline resectable pancreatic tumours typically undergo 6–12 weeks of neoadjuvant therapy before surgery. This period represents a logical point of intervention for obesity prehabilitation strategies [30]. Such integration may not only improve physical conditioning but also mitigate some of the treatment-induced metabolic derangements associated with chemotherapy or chemoradiation. While not directly examined in this setting, the interventions reported in this review are broadly compatible with neoadjuvant timelines and warrant prospective investigation.

The role of exercise-based prehabilitation also merits further study. Trials such as CHALLENGE [31] have already demonstrated the oncologic and functional benefits of structured physical activity in resected colon cancer. Extending such frameworks to the preoperative period in patients with obesity may offer the dual benefit of improved metabolic health and enhanced physical resilience at time of surgery [32].

This review has several limitations. All included studies were observational and thus subject to selection bias and residual confounding. Most did not adjust for comorbidity, tumour burden, or baseline performance status. Outcome definitions were variable, and complication severity grading was inconsistently reported. In addition, feasibility conclusions must be interpreted in context. Exercise protocols recommending up to 60 min per day may be unrealistic for some patients, particularly those with mobility limitations, concurrent chemotherapy, or baseline sarcopenia. Similarly, only a minority of studies enrolled patients undergoing neoadjuvant therapy, and the impact of concurrent cytotoxic treatment on adherence and efficacy remains underexplored. Wide generalisability is also limited. Six of the eight weight loss cohorts were from Japan, where baseline BMI is lower, patient support structures differ, and the applicability to Western populations with higher rates of severe obesity (BMI ≥ 40 or > 35 with obesity-related comorbidities) may be constrained.

Between-study baseline risk also varied widely (control-group complications ~ 13–70%; control-group leak rate ~ 0–17%), suggesting clinical heterogeneity not fully reflected by I². From a statistical perspective, anastomotic leak events were rare and several cohorts were unbalanced. We therefore used the Peto fixed-effect method for the primary leak analysis; however, Peto assumes balanced allocation and may be biased when this assumption is violated. In sensitivity analyses using MH pooling with continuity correction, the direction of effect was similar but estimates were imprecise. For overall complications, sensitivity analyses using Hartung–Knapp adjustment and exclusion of the largest study attenuated statistical significance (HKSJ OR 0.37, 95% CI 0.12–1.16; largest-study–excluded OR 0.43, 95% CI 0.11–1.65), indicating that results are sensitive to model choice and limited information size. Exploratory site-specific analyses (CRC vs. gastric) produced overlapping but differently precise estimates: CRC showed significant reductions in both overall complications and leak with low heterogeneity, whereas gastric estimates were imprecise with substantial heterogeneity. With ≤ 2 studies per subgroup and sparse events, these results are underpowered and should be viewed as hypothesis-generating rather than confirmatory.

Despite these limitations, the consistent directionality of effect across studies, and the biological rationale for benefit, through reduced adiposity, improved glucose regulation and simplified operative fields, support the potential value of intentional preoperative weight loss in selected patients. While causality cannot be confirmed from retrospective data, the findings justify future prospective studies that rigorously assess safety, oncologic timing, and cost-effectiveness.

## Conclusion

Intentional preoperative weight loss appears both feasible and safe in patients with obesity undergoing gastrointestinal or hepatobiliary cancer surgery. Across a range of interventions, high adherence and minimal treatment-related risk were observed, with no reported delays to definitive therapy. Where studied, weight loss was associated with a reduction in overall postoperative complications and anastomotic leak. These findings support the role of structured weight loss as a modifiable target within preoperative optimisation pathways, particularly in patients with central adiposity. The neoadjuvant window may provide an underused opportunity for such interventions. Prospective studies are needed to define optimal protocols, patient selection, and long-term oncologic safety, but current evidence supports careful implementation in selected patients.

## Supplementary Information

Below is the link to the electronic supplementary material.

Supplementary Material 1

## Data Availability

No datasets were generated or analysed during the current study.
