# Supplementary material for: Intentional Preoperative Weight Loss for Obesity in Patients Undergoing Gastrointestinal Cancer Resections: A Systematic Review and Meta-analysis
Source: J Gastrointest Cancer. 2026 Feb 3;57(1):31. doi: 10.1007/s12029-026-01405-1 (PMC12868102; doi:10.1007/s12029-026-01405-1)
Supplement: Supplementary file 1 — Supplementary Material 1 [file 12029_2026_1405_MOESM1_ESM.docx]

# Supplementary Material

Intentional pre-operative weight loss for obesity in patients undergoing gastrointestinal cancer resections: A systematic review and meta-analysis

**Authors**

Rathin Gosavi^1,2*^, Mehri Rasooli^3,4*^, Noel Leon^3,4^, Kousitha Sivayogan^3,4^, Dion Koh^3^, Jason Hong^3^, Vignesh Narasimhan^1,2,4^, Geraldine Ooi^3,4^

*co-first author

^1^Colorectal Unit, Monash Health, Clayton, Victoria, Australia

^2^Colorectal Unit, Cabrini Hospital

^3^Upper Gastrointestinal Unit, Monash Health, Clayton, Victoria, Australia.

^4^School of Clinical Sciences, Monash University, Clayton, Victoria, Australia.

**Corresponding Author**

Dr Geraldine Ooi, MBBS, BMedSc, PhD, FRACS

Department of Surgery, School of Clinical Sciences,

Faculty of Medicine, Nursing and Health Sciences Monash University, Victoria, 3168, Australia

T: +61 3 9594 6207

Email: geraldine.ooi@monash.edu

Supplementary Figure 1: Sensitivity analysis of overall complications using Hartung–Knapp–Sidik–Jonkman (HKSJ) adjustment.


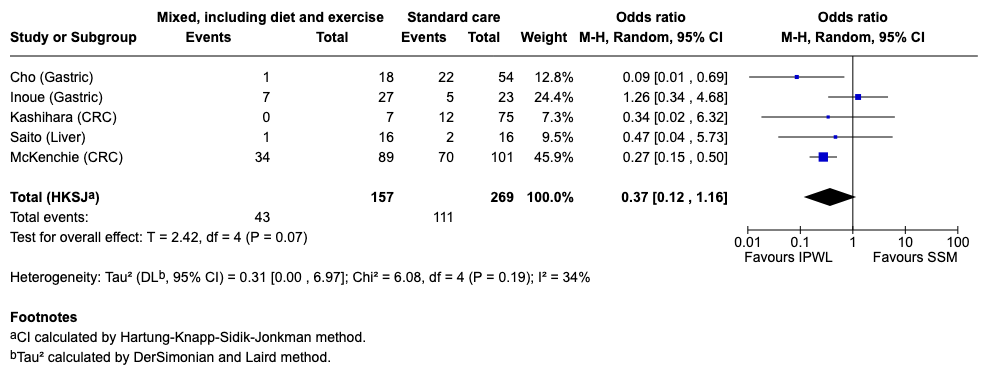


Supplementary Figure 2: Sensitivity analysis of overall complications excluding largest study (McKechnie et al.)


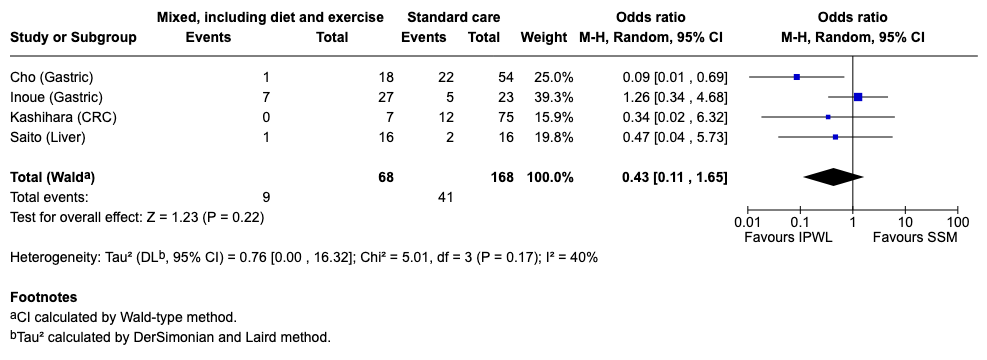


Supplementary Figure 3: Overall postoperative complication by cancer type


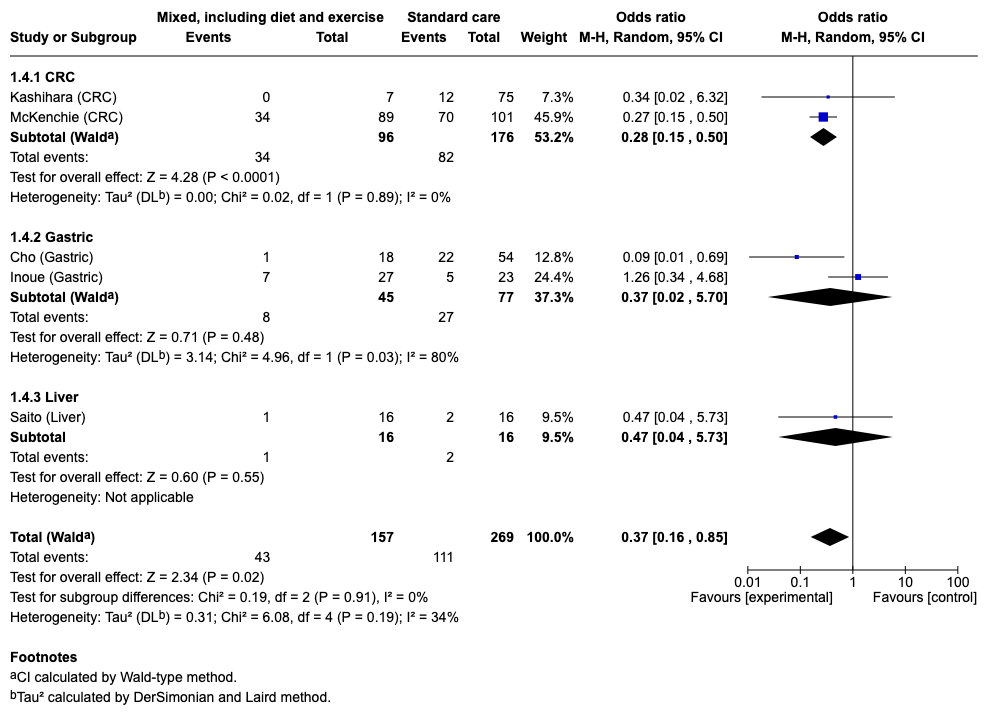


Supplementary Figure 4: Anastomotic leak by cancer type


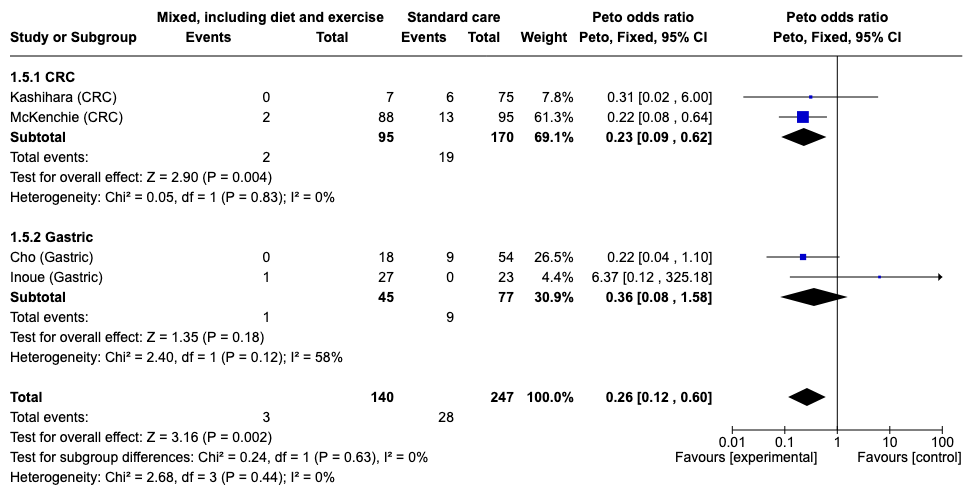


Supplementary Figure 5: Estimated blood loss – Weight loss intervention vs control
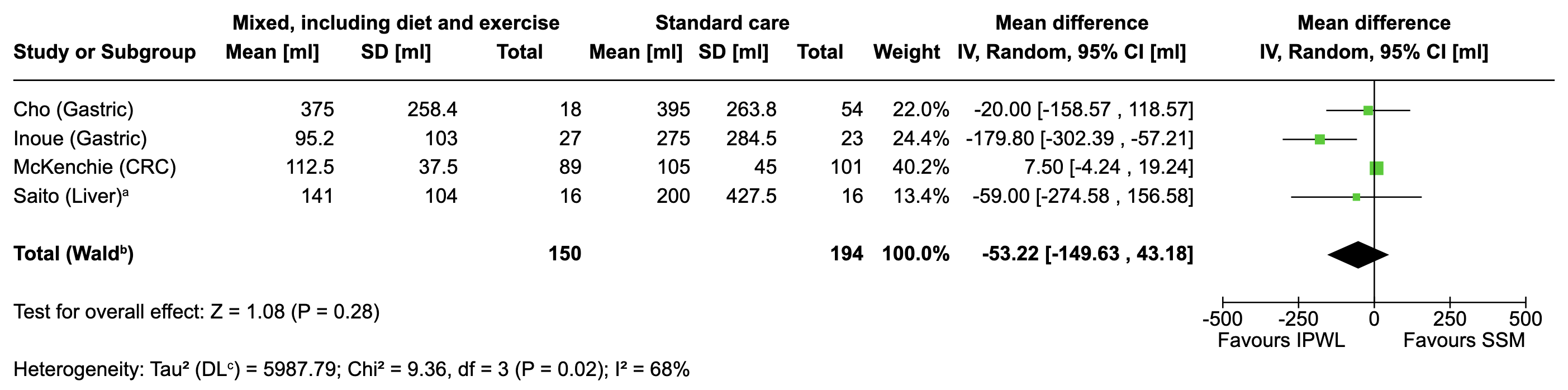


Supplementary Figure 6: Operative time – Weight loss intervention vs control


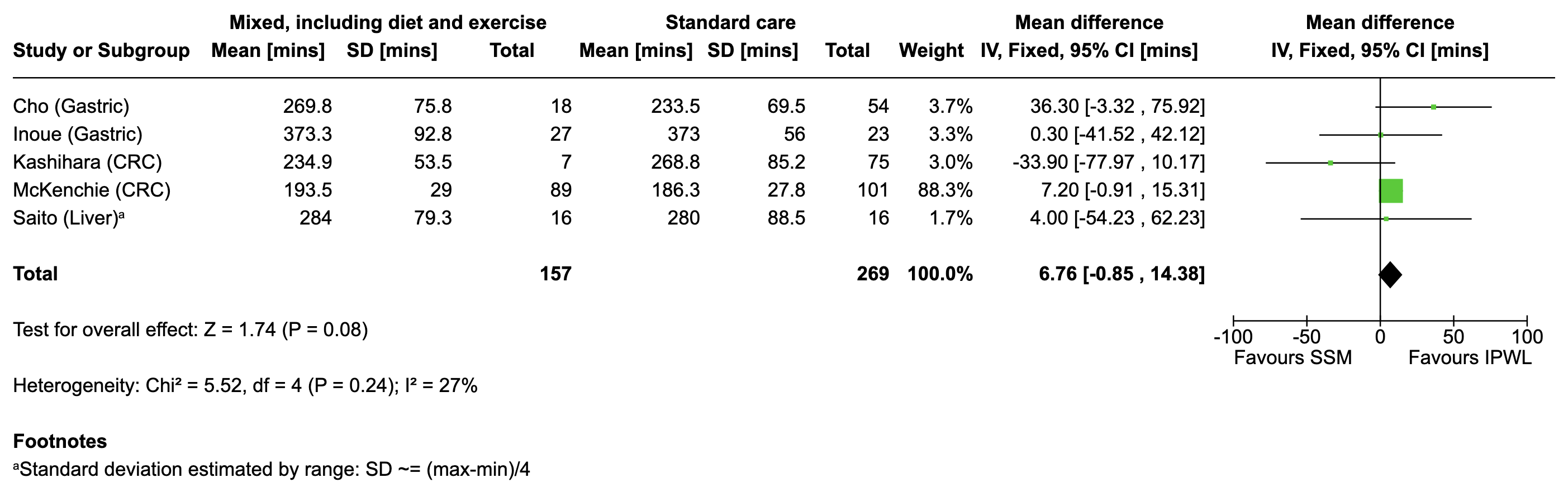


Supplementary Figure 7: Operative time by cancer type


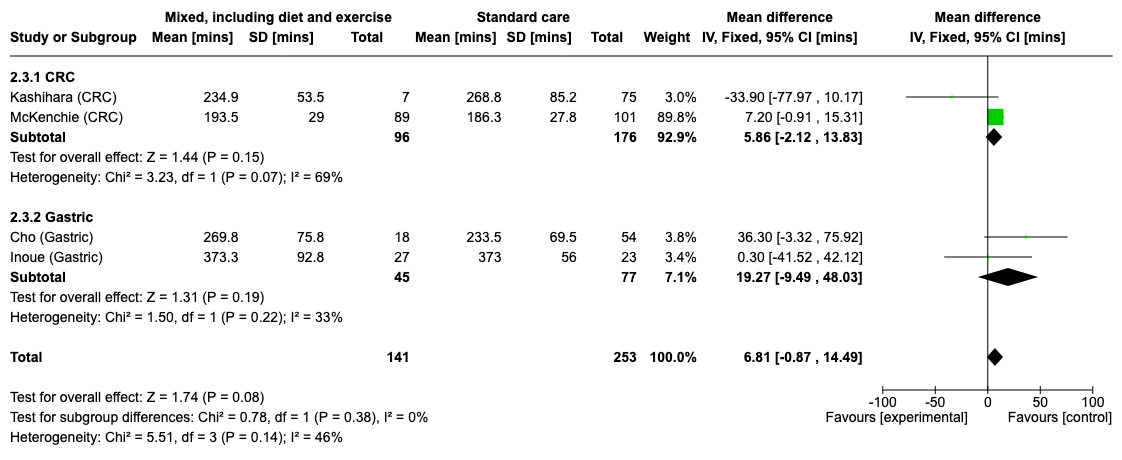


# Search Strategy

## Oesophageal cancer

### Medline Ovid

(obese or obesity or "high BMI" or "high body mass index" or overweight).mp.

exp Obesity/

(neoplasm or neoplasia or cancer).mp.

*esophagus/ or *esophageal/

exp Esophageal Neoplasms/

*esophagectomy/ or "*esophageal surgery".mp. or "*esophageal resection".mp. or "Ivor Lewis".mp. or "McKeown".mp. or "Sweet procedure".mp. or *esophagogastrectomy/

exp Esophagectomy/

exp Weight Loss/

exp Obesity Management/

exp Anti-Obesity Agents/

exp Diet, Ketogenic/ or exp Diet, High-Protein Low-Carbohydrate/ or exp Diet, Fat-Restricted/ or exp Diet Therapy/ or exp Diet, Carbohydrate-Restricted/ or exp Diet, Reducing/ or exp Diet, Healthy/ or exp Caloric Restriction/

exp Preoperative Exercise/ or exp Exercise/ or exp Exercise Therapy/

(VLCD or "low calorie diet" or "restricted diet" or "healthy diet" or "energy deficit diet" or "weight reduction").mp.

(physical activity or exercise).mp.

weight loss medication.mp.

(GLP-1 or semaglutide or tirzepatide or liraglutide or phentermine or orlistat or topiramate or metformin or "SGLT-2 inhibitors" or naltrexone or bupropion or lorcaserin or diethylpropion or "gelesis 100" or plenity or setmelanotide).mp.

1 or 2

(3 and 4) or 5 or 6 or 7

8 or 9 or 10 or 11 or 12 or 13 or 14 or 15 or 16

17 and 18 and 19

### Embase OVID

(obese or obesity or "high BMI" or "high body mass index" or overweight).mp.

exp Obesity/

(neoplasm or neoplasia or cancer).mp.

*esophagus/ or *esophageal/

exp Esophageal Neoplasms/

*esophagectomy/ or "*esophageal surgery".mp. or "*esophageal resection".mp. or "Ivor Lewis".mp. or "McKeown".mp. or "Sweet procedure".mp. or *esophagogastrectomy/

exp Esophagectomy/

exp Weight Loss/

exp Obesity Management/

exp Anti-Obesity Agents/

exp Diet, Ketogenic/ or exp Diet, High-Protein Low-Carbohydrate/ or exp Diet, Fat-Restricted/ or exp Diet Therapy/ or exp Diet, Carbohydrate-Restricted/ or exp Diet, Reducing/ or exp Diet, Healthy/ or exp Caloric Restriction/

exp Preoperative Exercise/ or exp Exercise/ or exp Exercise Therapy/

(VLCD or "low calorie diet" or "restricted diet" or "healthy diet" or "energy deficit diet" or "weight reduction").mp.

(physical activity or exercise).mp.

weight loss medication.mp.

(GLP-1 or semaglutide or tirzepatide or liraglutide or phentermine or orlistat or topiramate or metformin or "SGLT-2 inhibitors" or naltrexone or bupropion or lorcaserin or diethylpropion or "gelesis 100" or plenity or setmelanotide).mp.

1 or 2

(3 and 4) or 5 or 6 or 7

8 or 9 or 10 or 11 or 12 or 13 or 14 or 15 or 16

17 and 18 and 19

### Pubmed

obesity or obese or "high BMI" or “high body mass index” OR overweight

AND

((?esophageal cancer OR ?esophageal neoplas* OR “cancer ?esophagus”[tiab:~2] OR “neoplasm ?esophagus”[tiab:~2] OR “neoplasia ?esophagus”[tiab:~2]) AND (surgery OR resection OR operation)) OR (?esophagectomy OR ?esophageal surgery OR ?esophageal resection OR Ivor Lewis OR McKeown OR "Sweet procedure" or ?esophagogastrectomy)

AND

((weight loss OR weight optimi*ation OR weight management OR obesity management OR optimi*ation of obesity OR optimi*ation of weight ~~OR bariatric surgery OR bariatric procedure~~ OR VLCD OR “low calorie” OR “weight reduction” OR “calorie restriction” OR “caloric restriction” or “energy restriction” OR GLP-1 OR semaglutide OR tirzepatide OR liraglutide OR anti-obesity OR antiobesity))

AND

Pre-operative OR preoperative OR prehabilitation OR “Prior ?esophagectomy”[tiab:~2] OR “prior ?esophageal surgery”[tiab:~2] OR “prior surgery”[tiab:~2] or “prior resection”[tiab:~2] OR “prior ?esophageal resection” [tiab:~2] OR “prior Ivor Lewis” [tiab:~2] OR “prior McKeown” [tiab:~2] OR “prior Sweet procedure” [tiab:~2] or “prior ?oesophagectomy” [tiab:~2] OR “before ?esophagectomy”[tiab:~2] OR “before ?esophageal surgery”[tiab:~2] OR “before surgery”[tiab:~2] or “before resection”[tiab:~2] OR “before ?esophageal resection” [tiab:~2] OR “before Ivor Lewis” [tiab:~2] OR “before McKeown” [tiab:~2] OR “before Sweet procedure” [tiab:~2] or “before ?oesophagectomy” [tiab:~2]

### Cochrane

obesity OR obese OR (high NEXT BMI) OR (high body mass index) OR overweight

AND

(weight loss OR weight optimi*ation OR weight management OR obesity management OR optimi*ation of obesity OR optimi*ation of weight OR bariatric surgery OR bariatric procedure OR VLCD OR very low calorie OR weight reduction OR GLP-1 OR semaglutide OR tirzepatide OR liraglutide OR anti-obesity OR antiobesity):ti,ab,kw

AND

(((?esophageal cancer OR ?esophageal neoplas* OR cancer of the ?esophagus OR neoplas* of the ?esophagus) AND (surgery OR resection OR operation)) OR ?esophagectomy OR ?esophageal surgery OR ?esophageal resection OR Ivor Lewis OR McKeown OR Sweet procedure or ?esophagogastrectomy):ti,ab,kw

AND

AND

((prior OR before) AND (?esophagectomy OR ?esophageal surgery OR surgery OR resection OR ?esophageal resection OR Ivor Lewis OR McKeown OR "Sweet procedure" or ?esophagogastrectomy)) OR preoperative OR pre-operative OR prehabilitation

## Gastric cancer

### Medline Ovid

(obese or obesity or "high BMI" or "high body mass index" or overweight).mp.

exp Obesity/

(neoplasm or neoplasia or cancer).mp. and (exp Stomach/ or (stomach or gastric).mp.)

(gastrectomy or gastric surgery or stomach surgery or gastric resection or stomach resection or surgery or operation).mp. or exp Gastrectomy/

exp Weight Loss/ or exp Obesity Management/ or exp Bariatric Surgery/ or exp Anti-Obesity Agents/ or exp Diet/ or exp Exercise/ or (VLCD or "low calorie diet" or "restricted diet" or "healthy diet" or "energy deficit diet" or "weight reduction" or physical activity or exercise or weight loss medication or GLP-1 or semaglutide or tirzepatide or liraglutide or phentermine or orlistat or topiramate or metformin or SGLT-2 or naltrexone or bupropion or lorcaserin or diethylpropion or gelesis 100 or plenity or setmelanotide).mp.

(1 or 2) and 3 and 4 and 5

exp Preoperative Exercise/ or preoperative.mp. or pre-operative.mp. or exp Preoperative Care/ or exp Preoperative Period/ or prehabilitation.mp. or prehabilitative.mp. or pre-surg*.mp. or presurg*.mp.

6 and 7

### Embase OVID

(obese or obesity or "high BMI" or "high body mass index" or overweight).mp.

exp Obesity/

(neoplasm or neoplasia or cancer).mp. and (exp Stomach/ or (stomach or gastric).mp.)

(gastrectomy or gastric surgery or stomach surgery or gastric resection or stomach resection or surgery or operation).mp. or exp Gastrectomy/

exp Weight Loss/ or exp Obesity Management/ or exp Bariatric Surgery/ or exp Anti-Obesity Agents/ or exp Diet/ or exp Exercise/ or (VLCD or "low calorie diet" or "restricted diet" or "healthy diet" or "energy deficit diet" or "weight reduction" or physical activity or exercise or weight loss medication or GLP-1 or semaglutide or tirzepatide or liraglutide or phentermine or orlistat or topiramate or metformin or SGLT-2 or naltrexone or bupropion or lorcaserin or diethylpropion or gelesis 100 or plenity or setmelanotide).mp.

(1 or 2) and 3 and 4 and 5

exp Preoperative Exercise/ or preoperative.mp. or pre-operative.mp. or exp Preoperative Care/ or exp Preoperative Period/ or prehabilitation.mp. or prehabilitative.mp. or pre-surg*.mp. or presurg*.mp.

6 and 7

### Pubmed

obesity or obese or "high BMI" or “high body mass index” or overweight

AND

(gastric cancer OR gastric neoplas* OR stomach cancer or stomach neoplas* OR cancer of the stomach OR neoplas* of the stomach) AND (surgery OR resection OR operation OR gastrectomy OR gastric surgery OR gastric resection OR stomach surgery OR stomach resection)

AND

"weight loss" OR "weight optimisation" OR "weight optimization" OR "weight management" OR "obesity management" OR "optimization of obesity" OR "optimisation of obesity" OR "optimization of weight" OR "optimisation of weight" OR VLCD OR "low calorie" OR "weight reduction" OR "calorie restriction" OR "caloric restriction" or "energy restriction" OR GLP-1 OR semaglutide OR tirzepatide OR liraglutide OR anti-obesity OR antiobesity

AND

preoperative OR pre-operative or prehabilitation or prehabilitative or pre-surg* or presurg*

### Cochrane

obesity OR obese OR (high NEXT BMI) OR (high body mass index) OR overweight

AND

weight loss OR weight reduction OR weight optimisation OR weight optimization OR obesity management OR obesity optimisation OR obesity optimization

AND

(gastric OR stomach) AND (cancer OR neoplasia OR neoplasm) AND (gastrectomy OR surgery OR resection OR operation)

obesity or obese or "high BMI" or overweight

AND

(stomach cancer OR gastric cancer OR stomach neoplas* OR gastric neoplas* OR cancer of the stomach OR neoplas* of the stomach) AND (gastrectomy OR surgery OR resection OR operation)

AND

("weight loss" OR "weight optimi*ation" OR "weight management" OR "obesity management" OR "optimi*ation of obesity" OR "optimi*ation of weight" OR "bariatric surgery" OR "bariatric procedure" OR VLCD OR "very low calorie" OR "weight reduction" OR GLP-1 OR semaglutide OR tirzepatide OR liraglutide OR anti-obesity OR antiobesity) AND (pre-operative OR before surgery OR preoperative OR perioperative OR peri-operative OR "prior to surgery" OR "prior to resection" OR "prior to operation" OR "followed by surgery" OR "followed by operation" OR "followed by resection")

## Colon and rectal cancer

### Medline Ovid

(obese or obesity or "high BMI" or "high body mass index" or overweight).mp.

exp Obesity/

1 or 2

(neoplasm or neoplasia or cancer).mp. or exp Neoplasms/

exp Colon/ or (colon or colonic or rectum or rectal or colorectal).mp. or exp Rectum/

4 and 5

exp Colorectal Neoplasms/

6 or 7

exp Colorectal Surgery/ or (colectomy or colon surgery or colonic surgery or rectal surgery or rectum surgery or colon resection or colonic resection or rectal resection or rectum resection or surgery or operation or resection or anterior resection or abdominoperineal resection or hemicolectomy or pelvic exenteration).mp.

exp Weight Loss/ or exp Obesity Management/ or exp Bariatric Surgery/ or exp Anti-Obesity Agents/ or exp Diet/ or exp Exercise/ or (VLCD or "low calorie diet" or "restricted diet" or "healthy diet" or "energy deficit diet" or "weight reduction" or physical activity or exercise or weight loss medication or GLP-1 or semaglutide or tirzepatide or liraglutide or phentermine or orlistat or topiramate or metformin or SGLT-2 or naltrexone or bupropion or lorcaserin or diethylpropion or gelesis 100 or plenity or setmelanotide).mp.

3 and 8 and 9 and 10

exp Preoperative Exercise/ or preoperative.mp. or pre-operative.mp. or exp Preoperative Care/ or exp Preoperative Period/ or prehabilitation.mp. or prehabilitative.mp. or pre-surg*.mp. or presurg*.mp.

11 and 12

### Embase OVID

(obese or obesity or "high BMI" or "high body mass index" or overweight).mp.

exp Obesity/

1 or 2

(neoplasm or neoplasia or cancer).mp. or exp Neoplasms/

exp Colon/ or (colon or colonic or rectum or rectal or colorectal).mp. or exp Rectum/

4 and 5

exp Colorectal Neoplasms/

6 or 7

exp Colorectal Surgery/ or (colectomy or colon surgery or colonic surgery or rectal surgery or rectum surgery or colon resection or colonic resection or rectal resection or rectum resection or surgery or operation or resection or anterior resection or abdominoperineal resection or hemicolectomy or pelvic exenteration).mp.

exp Weight Loss/ or exp Obesity Management/ or exp Bariatric Surgery/ or exp Anti-Obesity Agents/ or exp Diet/ or exp Exercise/ or (VLCD or "low calorie diet" or "restricted diet" or "healthy diet" or "energy deficit diet" or "weight reduction" or physical activity or exercise or weight loss medication or GLP-1 or semaglutide or tirzepatide or liraglutide or phentermine or orlistat or topiramate or metformin or SGLT-2 or naltrexone or bupropion or lorcaserin or diethylpropion or gelesis 100 or plenity or setmelanotide).mp.

3 and 8 and 9 and 10

exp Preoperative Exercise/ or preoperative.mp. or pre-operative.mp. or exp Preoperative Care/ or exp Preoperative Period/ or prehabilitation.mp. or prehabilitative.mp. or pre-surg*.mp. or presurg*.mp.

11 and 12

### Pubmed

obesity or obese or "high BMI" or “high body mass index” OR overweight

AND

“weight loss” OR “weight optimi*ation” OR “weight management” OR “obesity management” OR “optimi*ation obesity”[tiab:~1] OR “optimi*ation weight”[tiab:~1] OR “management weight”[tiab:~1] OR “management obesity”[tiab:~1] OR bariatric surgery OR bariatric procedure OR VLCD OR “low calorie” OR “weight reduction” OR “calorie restriction” OR “caloric restriction” or “energy restriction” OR GLP-1 OR semaglutide OR tirzepatide OR liraglutide OR anti-obesity OR antiobesity

AND

Pre-operative OR preoperative OR prehabilitation OR “Prior to ?colectomy” OR “prior to colon* surgery” OR “prior to rect* surgery” OR “prior to surgery” or “prior to resection” OR “prior to colon* resection” OR “prior to rect* resection” OR “prior to abdominoperineal resection” OR “prior to APR” OR “prior pelvic exenteration” OR “before ?colectomy” OR “before colon* surgery” OR “before rect* surgery” OR “before surgery” or “before resection” OR “before colon* resection” OR “before rect* resection” OR “before APR” OR “before abdominoperineal resection” OR “before pelvic exenteration”

AND

("colon* cancer" or “rect* cancer” OR “anal cancer” OR "colon* neoplas*" or “rect* neoplas*” or "anal neoplas*" OR "cancer of the colon" OR "neoplas* of the colon" or "neoplas* of the rectum" OR "cancer of the rectum" OR “cancer of the anus” or “neoplas* of the anus”) AND (surgery OR resection OR operation OR colectomy OR hemicolectomy OR proctocolectomy or “anterior resection” OR APR or “abdominoperineal resection” OR "colon* surgery" OR “rectal surgery” or "colon* resection" OR “anal surgery” OR “rectal resection” or “pelvic exenteration”)

### Cochrane

obesity OR obese OR (high NEXT BMI) OR (high body mass index) OR overweight

AND

weight loss OR weight reduction OR weight optimisation OR weight optimization OR obesity management OR obesity optimisation OR obesity optimization

AND

(colon OR rectum OR anus OR rectal OR anal OR colonic OR colorectal) AND (cancer OR neoplasia OR neoplasm) AND (colectomy OR APR OR surgery OR resection OR operation)

## Liver resections

### Medline Ovid

1. (obese or obesity or "high BMI" or "high body mass index" or overweight).mp. or exp Obesity/

2. (neoplasm or neoplasia or cancer or metastasis or metastases).mp. or exp Neoplasms/

3. exp Liver/ or (liver or hepatic).mp.

4. 2 and 3

5. exp Liver Neoplasms/ or exp Carcinoma, Hepatocellular/ or (hepatocellular carcinoma or HCC).mp.

6. 4 or 5

7. exp Hepatectomy/ or (hepatectomy or hemihepatectomy or liver surgery or hepatic surgery or liver resection or hepatic resection or operation or resection).mp.

8. exp Weight Loss/ or exp Obesity Management/ or exp Bariatric Surgery/ or exp Anti-Obesity Agents/ or exp Diet/ or exp Exercise/ or (weight loss or obesity management or VLCD or "low calorie diet" or "restricted diet" or "healthy diet" or "energy deficit diet" or "weight reduction" or physical activity or exercise or weight loss medication or GLP-1 or semaglutide or tirzepatide or liraglutide or phentermine or orlistat or topiramate or metformin or SGLT-2 or naltrexone or bupropion or lorcaserin or diethylpropion or gelesis 100 or plenity or setmelanotide).mp.

9. 1 and 6 and 7 and 8

### Embase OVID

1. (obese or obesity or "high BMI" or "high body mass index" or overweight).mp. or exp Obesity/

2. (neoplasm or neoplasia or cancer or metastasis or metastases).mp. or exp Neoplasms/

3. exp Liver/ or (liver or hepatic).mp.

4. 2 and 3

5. exp Liver Neoplasms/ or exp Carcinoma, Hepatocellular/ or (hepatocellular carcinoma or HCC).mp.

6. 4 or 5

7. exp Hepatectomy/ or (hepatectomy or hemihepatectomy or liver surgery or hepatic surgery or liver resection or hepatic resection or operation or resection).mp.

8. exp Weight Loss/ or exp Obesity Management/ or exp Bariatric Surgery/ or exp Anti-Obesity Agents/ or exp Diet/ or exp Exercise/ or (weight loss or obesity management or VLCD or "low calorie diet" or "restricted diet" or "healthy diet" or "energy deficit diet" or "weight reduction" or physical activity or exercise or weight loss medication or GLP-1 or semaglutide or tirzepatide or liraglutide or phentermine or orlistat or topiramate or metformin or SGLT-2 or naltrexone or bupropion or lorcaserin or diethylpropion or gelesis 100 or plenity or setmelanotide).mp.

9. 1 and 6 and 7 and 8

### Pubmed

obesity or obese or "high BMI" or “high body mass index” OR overweight

AND

"weight loss" OR "weight optimisation" OR "weight optimization" OR "weight management" OR "obesity management" OR "obesity optimi*ation" or "weight optimi*ation" OR "optimization of obesity" OR "optimisation of obesity" OR "optimization of weight" OR "optimisation of weight" OR VLCD OR "low calorie" OR "weight reduction" OR "calorie restriction" OR "caloric restriction" or "energy restriction" OR GLP-1 OR semaglutide OR tirzepatide OR liraglutide OR anti-obesity OR antiobesity

AND

Pre-operative OR preoperative OR prehabilitation OR “Prior to ?hepatectomy” OR “prior to liver surgery” OR “prior to hepatic surgery” OR “prior to surgery” or “prior to resection” OR “prior to liver resection” OR “prior to hepatic resection” OR “before ?hepatectomy” OR “before liver surgery” OR “before hepatic surgery” OR “before surgery” or “before resection” OR “before liver resection” OR “before hepatic resection”

AND

("liver cancer" or “hepatic cancer” OR "liver neoplas*" or “hepatic neoplas*” OR "cancer of the liver" OR "neoplas* of the liver" or “liver metastasis” or “liver metastases” or “hepatic metastasis” or “hepatic metastases” OR “hepatic malignancy” OR “liver malignancy” OR “hepatocellular carcinoma” or HCC) AND (surgery OR resection OR operation OR hepatectomy OR hemihepatectomy OR "liver surgery" OR “hepatic surgery” or "liver resection" OR “hepatic resection”)

### Cochrane

obesity OR obese OR (high NEXT BMI) OR (high body mass index) OR overweight

AND

weight loss OR weight reduction OR weight optimisation OR weight optimization OR obesity management OR obesity optimisation OR obesity optimization

AND

(liver OR hepatic) AND (cancer OR neoplasia OR neoplasm) AND (hepatectomy OR hemihepatectomy OR surgery OR resection OR operation OR segmentectomy OR sectionectomy)

## Biliary/gallbladder cancer

### Medline Ovid

1. (obese or obesity or "high BMI" or "high body mass index" or overweight).mp. or exp Obesity/

2. (neoplasm or neoplasia or cancer or malignancy or malignant or carcinoma or adenocarcinoma).mp. or exp Neoplasms/

3. exp Bile Ducts/ or (bile duct or biliary or gallbladder).mp. or exp Gallbladder/

4. 2 and 3

5. exp Gallbladder Neoplasms/ or exp Cholangiocarcinoma/ or exp Bile Duct Neoplasms/ or cholangiocarcinoma.mp.

6. 4 or 5

7. exp Biliary Tract Surgical Procedures/ or exp Cholecystectomy/ or (bile duct excision or bile duct resection or bile duct operation or cholecystectomy or hepaticojejunostomy or operation or surgery or resection or excision).mp.

8. exp Weight Loss/ or exp Obesity Management/ or exp Bariatric Surgery/ or exp Anti-Obesity Agents/ or exp Diet/ or exp Exercise/ or (weight loss or obesity management or VLCD or "low calorie diet" or "restricted diet" or "healthy diet" or "energy deficit diet" or "weight reduction" or physical activity or exercise or weight loss medication or GLP-1 or semaglutide or tirzepatide or liraglutide or phentermine or orlistat or topiramate or metformin or SGLT-2 or naltrexone or bupropion or lorcaserin or diethylpropion or gelesis 100 or plenity or setmelanotide).mp.

9. 1 and 6 and 7 and 8

### Embase OVID

1. (obese or obesity or "high BMI" or "high body mass index" or overweight).mp. or exp Obesity/

2. (neoplasm or neoplasia or cancer or malignancy or malignant or carcinoma or adenocarcinoma).mp. or exp Neoplasms/

3. exp Bile Ducts/ or (bile duct or biliary or gallbladder).mp. or exp Gallbladder/

4. 2 and 3

5. exp Gallbladder Neoplasms/ or exp Cholangiocarcinoma/ or exp Bile Duct Neoplasms/ or cholangiocarcinoma.mp.

6. 4 or 5

7. exp Biliary Tract Surgical Procedures/ or exp Cholecystectomy/ or (bile duct excision or bile duct resection or bile duct operation or cholecystectomy or hepaticojejunostomy or operation or surgery or resection or excision).mp.

8. exp Weight Loss/ or exp Obesity Management/ or exp Bariatric Surgery/ or exp Anti-Obesity Agents/ or exp Diet/ or exp Exercise/ or (weight loss or obesity management or VLCD or "low calorie diet" or "restricted diet" or "healthy diet" or "energy deficit diet" or "weight reduction" or physical activity or exercise or weight loss medication or GLP-1 or semaglutide or tirzepatide or liraglutide or phentermine or orlistat or topiramate or metformin or SGLT-2 or naltrexone or bupropion or lorcaserin or diethylpropion or gelesis 100 or plenity or setmelanotide).mp.

9. 1 and 6 and 7 and 8

### Pubmed

obesity or obese or "high BMI" or “high body mass index” OR overweight

AND

"weight loss" OR "weight optimisation" OR "weight optimization" OR "weight management" OR "obesity management" OR "obesity optimi*ation" or "weight optimi*ation" OR "optimization of obesity" OR "optimisation of obesity" OR "optimization of weight" OR "optimisation of weight" OR VLCD OR "low calorie" OR "weight reduction" OR "calorie restriction" OR "caloric restriction" or "energy restriction" OR GLP-1 OR semaglutide OR tirzepatide OR liraglutide OR anti-obesity OR antiobesity

AND

Pre-operative OR preoperative OR prehabilitation OR “prior to hepaticojejunostomy” OR “prior to cholecystectomy” OR “prior to bile duct surgery” OR “prior to biliary surgery” OR “prior to gallbladder surgery” OR “prior to surgery” or “prior to resection” OR “prior to bile duct resection” OR “prior to biliary resection” OR “prior to gallbladder resection” OR “before cholecystectomy” OR “before gallbladder surgery” OR “before bile duct surgery” OR “before biliary surgery” OR “before surgery” or “before resection” OR “before bile duct resection” OR “before biliary resection”

AND

("bile duct cancer" or “biliary cancer” OR “gallbladder cancer” OR "bile duct neoplas*" or “biliary neoplas*” OR “gallbladder neoplas*” OR “bile duct malignancy” OR “biliary malignancy” OR “gallbladder malignancy” OR "cancer of the bile duct" OR “cancer of the gallbladder” OR "neoplas* of the bile duct" OR “neoplas* of the gallbladder” OR cholangiocarcinoma or bile duct adenocarcinoma or biliary adenocarcinoma or gallbladder adenocarcinoma)

### Cochrane

obesity OR obese OR (high NEXT BMI) OR (high body mass index) OR overweight

AND

weight loss OR weight reduction OR weight optimisation OR weight optimization OR obesity management OR obesity optimisation OR obesity optimization

AND

((biliary OR bile duct OR gallbladder) AND (cancer OR neoplasia OR neoplasm OR adenocarcinoma OR cholangiocarcinoma OR malignancy)) or (cholangiocarcinoma)

AND

(surgery OR resection OR operation)

## Pancreas cancer

### Medline Ovid

| 1. (obese or obesity or "high BMI" or "high body mass index" or overweight).mp. or exp Obesity/ |
| --- |

2. (neoplasm or neoplasia or cancer or malignancy or malignant or tumour or tumor or carcinoma or adenocarcinoma).mp. or exp Neoplasms/

3. exp Pancreas/ or (pancreas or pancreatic).mp.

4. 2 and 3

5. exp Pancreatic Neoplasms/ or (pancreatic cancer or pancreas cancer or pancreatic malignancy or pancreas malignancy or pancreatic tumour or pancreas tumour or pancreatic tumor or pancreas tumor).mp.

6. 4 or 5

7. exp Pancreatectomy/ or exp Pancreaticoduodenectomy/ or (pancreatectomy or pancreaticoduodenectomy or Whipple or operation or surgery or resection or excision).mp.

8. exp Weight Loss/ or exp Obesity Management/ or exp Bariatric Surgery/ or exp Anti-Obesity Agents/ or exp Diet/ or exp Exercise/ or (weight loss or obesity management or VLCD or "low calorie diet" or "restricted diet" or "healthy diet" or "energy deficit diet" or "weight reduction" or physical activity or exercise or weight loss medication or GLP-1 or semaglutide or tirzepatide or liraglutide or phentermine or orlistat or topiramate or metformin or SGLT-2 or naltrexone or bupropion or lorcaserin or diethylpropion or gelesis 100 or plenity or setmelanotide).mp.

9. 1 and 6 and 7 and 8

### Embase OVID

1. (obese or obesity or "high BMI" or "high body mass index" or overweight).mp. or exp Obesity/

2. (neoplasm or neoplasia or cancer or malignancy or malignant or tumour or tumor or carcinoma or adenocarcinoma).mp. or exp Neoplasms/

3. exp Pancreas/ or (pancreas or pancreatic).mp.

4. 2 and 3

5. exp Pancreatic Neoplasms/ or (pancreatic cancer or pancreas cancer or pancreatic malignancy or pancreas malignancy or pancreatic tumour or pancreas tumour or pancreatic tumor or pancreas tumor).mp.

6. 4 or 5

7. exp Pancreatectomy/ or exp Pancreaticoduodenectomy/ or (pancreatectomy or pancreaticoduodenectomy or Whipple or operation or surgery or resection or excision).mp.

8. exp Weight Loss/ or exp Obesity Management/ or exp Bariatric Surgery/ or exp Anti-Obesity Agents/ or exp Diet/ or exp Exercise/ or (weight loss or obesity management or VLCD or "low calorie diet" or "restricted diet" or "healthy diet" or "energy deficit diet" or "weight reduction" or physical activity or exercise or weight loss medication or GLP-1 or semaglutide or tirzepatide or liraglutide or phentermine or orlistat or topiramate or metformin or SGLT-2 or naltrexone or bupropion or lorcaserin or diethylpropion or gelesis 100 or plenity or setmelanotide).mp.

9. 1 and 6 and 7 and 8

### Pubmed

obesity or obese or "high BMI" or “high body mass index” OR overweight

AND

"weight loss" OR "weight optimisation" OR "weight optimization" OR "weight management" OR "obesity management" OR "obesity optimi*ation" or "weight optimi*ation" OR "optimization of obesity" OR "optimisation of obesity" OR "optimization of weight" OR "optimisation of weight" OR VLCD OR "low calorie" OR "weight reduction" OR "calorie restriction" OR "caloric restriction" or "energy restriction" OR GLP-1 OR semaglutide OR tirzepatide OR liraglutide OR anti-obesity OR antiobesity

AND

Pre-operative OR preoperative OR prehabilitation OR “Prior to pancreatectomy” OR “prior to pancreaticoduodenectomy” OR “prior to Whipple” OR “prior to pancreas surgery” OR “prior to pancreatic surgery” OR “prior to surgery” or “prior to resection” OR “prior to pancreas resection” OR “prior to pancreatic resection” OR “before pancreatectomy” OR “before pancreaticoduodenectomy” OR “before Whipple” OR “before pancreas surgery” OR “before pancreatic surgery” OR “before surgery” or “before resection” OR “before pancreas resection” OR “before pancreatic resection”

AND

("pancreas cancer" or “pancreatic cancer” OR "pancreas neoplas*" or “pancreatic neoplas*” OR “pancreas malignancy” OR “pancreatic malignancy” OR "cancer of the pancreas" OR "neoplas* of the pancreas" or pancreatic neuroendocrine tumour or pancreatic adenocarcinoma or pancreas adenocarcinoma or pNET or PDAC) AND (surgery OR resection OR operation OR pancreatectomy OR pancreaticoduodenectomy OR Whipple OR "pancreas surgery" OR “pancreatic surgery” or "pancreas resection" OR “pancreatic resection”)

### Cochrane

obesity OR obese OR (high NEXT BMI) OR (high body mass index) OR overweight

AND

weight loss OR weight reduction OR weight optimisation OR weight optimization OR obesity management OR obesity optimisation OR obesity optimization

AND

((pancreas OR pancreatic) AND (cancer OR neoplasia OR neoplasm OR adenocarcinoma OR neuroendocrine OR malignancy OR carcinoma)) or (PDAC or pNET)

AND

(surgery OR resection OR operation)
